# 4564 Nilotinib alters microRNAs that regulate specific autophagy and ubiquitination genes in the cerebrospinal fluid of Parkinson’s patients

**DOI:** 10.1017/cts.2020.308

**Published:** 2020-07-29

**Authors:** Alan Fowler, Yasar Torres-Yhagi, Fernando Pagan, Michaeline Hebron, Barbara Willmarth, Joy Arellano, Helen Howard, Sara Matar, Timothy Chiu, Jaeil Ahn, Charbel Moussa

**Affiliations:** 1 Georgetown - Howard Universities; 2 MedStar Georgetown University Hospital; 3 Georgetown University Medical Center

## Abstract

OBJECTIVES/GOALS: Our preclinical data demonstrate that the principal effects of nilotinib, a multi-tyrosine kinase inhibitor, in models of neurodegeneration is clearance of misfolded proteins via autophagy. Here we aimed to evaluate the effects of nilotinib on microRNAs in the cerebrospinal fluid of Parkinson’s disease patients. METHODS/STUDY POPULATION: Cerebrospinal fluid (CSF) was collected as part of an open label phase I (NCT02281474) (n = 12, 300 mg nilotinib taken orally once daily for 6 months), and a phase II randomized, double-blind, placebo-controlled study (NCT02954978) (n = 75, randomized 1:1:1 into placebo, 150 mg or 300 mg nilotinib taken orally once daily for 12 months). RNA was isolated from CSF and Indexed sequencing libraries were prepared from total RNA plus miRNA. Next generation whole-genome sequencing (single-end 1x75 bp, 25 million raw reads per sample) was performed to identify miRNAs significantly differentially expressed (fold-change ≥ 2, Benjamini-Hochberg FDR p-value ≤ 0.05 or Empirical Bayes FDR ≤ 0.05) with treatment compared to baseline. RESULTS/ANTICIPATED RESULTS: Next generation whole-genome sequencing of microRNAs in the CSF demonstrated that nilotinib significantly increases microRNAs that specifically regulate expression of autophagy and ubiquitination genes in individuals with Parkinson’s disease. In the open label phase I, samples, 28 microRNAs found to regulate autophagy and ubiquitination genes, were significantly altered with treatment (Benjamini-Hochberg FDR p-value ≤ 0.05). In the phase II randomized, double-blind, placebo-controlled study samples, we verified several of those 28 candidate microRNAs had been significantly deferentially expressed with treatment (Empirical Bayes FDR p-value ≤ 0.05). DISCUSSION/SIGNIFICANCE OF IMPACT: Our data provide robust evidence that nilotinib’s effects on misfolded protein clearance is via autophagy and CSF miRNA sequencing is a valid biomarker of nilotinib’s effects in a definitive phase III study to investigate nilotinib in Parkinson’s and other neurodegenerative diseases. CONFLICT OF INTEREST DESCRIPTION: Charbel Moussa is listed as an inventor on several Georgetown University patents for the use of tyrosine kinase inhibitors as a treatment for neurodegenerative diseases

